# High-speed X-ray ptychographic tomography

**DOI:** 10.1038/s41598-022-11292-8

**Published:** 2022-05-12

**Authors:** Darren Batey, Christoph Rau, Silvia Cipiccia

**Affiliations:** 1 Diamond Light Source, Harwell Science and Innovation Campus, Fermi Ave, Didcot, OX11 0DE UK; 2 Department of Medical Physics and Biomedical Engineering, University College London, Gower Street, London, WC1E 6BT UK

**Keywords:** Imaging techniques, Materials for energy and catalysis, Scanning probe microscopy

## Abstract

X-ray ptychography is a coherent scanning imaging technique widely used at synchrotron facilities for producing quantitative phase images beyond the resolution limit of conventional x-ray optics. The scanning nature of the technique introduces an inherent overhead to the collection at every scan position and limits the acquisition time of each 2D projection. The overhead associated with motion can be minimised with a continuous-scanning approach. Here we present an acquisition architecture based on continuous-scanning and up-triggering which allows to record ptychographic datasets at up to 9 kHz. We demonstrate the method by applying it to record 2D scans at up to 273 µm^2^/s and 3D scans of a (20 µm)^3^ volume in less than three hours. We discuss the current limitations and the outlook toward the development of sub-second 2D acquisition and minutes-long 3D ptychographic tomograms.

## Introduction

X-ray ptychography is a coherent scanning imaging technique that produces high-resolution quantitative phase imaging at the nanoscale^1^. A ptychographic acquisition consists of scanning a sample relative to a localised coherent spot of illumination, with subsequently exposed regions overlapping, while recording the diffraction pattern in the far field. The inversion of the diffraction pattern is performed using iterative algorithms^2–5^ which retrieve the complex functions of both the sample and the illumination. The technique requires no objective lens and is ultimately diffraction limited, unlocking imaging resolutions beyond the limit of conventional optics and detectors. Thanks to the unique capability to produce highly sensitive phase images at the highest resolutions, ptychography has become a go-to method across the X-ray synchrotron imaging community for nanoscale imaging. It has been successfully applied in different research fields, from energy to electronics and from magnetism to life science^6–8^. The acquisition time is limited by motion overheads due to the scanning nature. In the state-of-the-art step-scanning ptychography the overhead has been reduced to 5 ms^9^, bringing the maximum acquisition rate up to a 200 Hz limit. Further minimisation of these overheads is critical for increasing the throughput and extending the applications of ptychographic imaging. Much progress has already been made towards this goal with the successful implementation of continuous-scanning methods at the major synchrotrons around the world^10–17^, that has allowed for 2D acquisitions up to 3 kHz. In a continuous scanning approach, the sample is moved at a constant velocity while recording a series of diffraction patterns. The continuous scan approach reduces the overheads characteristic of the step-and-shot motion from the millisecond regime to the microsecond regime. The blurring effects introduced by the continuous motion, are alleviated by advanced retrieval algorithms.

We propose a data acquisition strategy exploiting the full capability of the detector recording rate of up to 9 kHz. We have applied the proposed approach to record and reconstruct a 9 kHz 2D scan and a 2 kHz 3D ptychographic tomography of a (20 µm)^3^ sample in less than three hours.

## Results

### kHz scanning acquisition scheme

The kHz continuous scanning system implemented at I13-1 is built up from a series of hardware components. The hardware chain consists of a GEO brick Turbo PMAC motion controller, which controls the motion of a 3-axis PI-Mars piezo stage and the triggering of a Zebra trigger box^18^, which triggers the detector and captures the motor positions. The motion controller executes low level PMAC scripts based on the scanning architecture developed at the I24 beamline at Diamond Light Source^19,20^. The scan parameters are configurable with a simple command line interface at the beamline. The motors are moved in a snake-like trajectory with continuous scanning in the horizontal axis and step scanning in the vertical. The motion controller allows for look-ahead calculations of the trajectory, where the block rate of the calculations depends on the number of motors involved. When controlling 3 motors, as for the implemented setup, the maximum block rate is 1 kHz. The motion controller generates trigger pulses as the sample passes through each of the regularly spaced positions requested, based on the calculated trajectory. The in-position trigger signal is sent to the Zebra trigger box which captures the encoder positions of the motors, storing them in a HDF file, and sends out an acquire signal to the detector.

Since ptychography does not require the acquisition points to be on a regular grid, it is possible to collect data at points in-between the in-position triggers sent by the motion controller. The Zebra trigger box is capable of generating trigger trains up to 3 kHz. The 1 kHz signal from the motion controller can be up-triggered within the Zebra box, by generating equally time-spaced pulses for each in-position trigger, up to 3 kHz. This enables the capture of data up to three times faster than the calculation and tracking rate of the motion controller.

**Figure 1:**
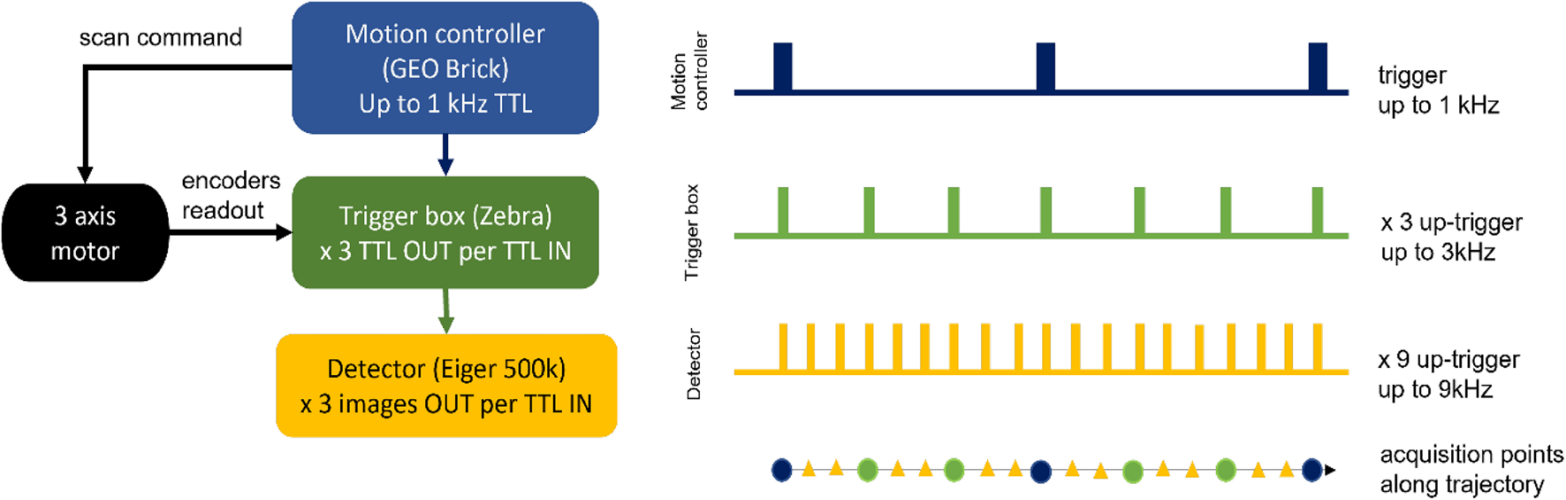
Schematic of the trigger chain which allows for up to 9 kHz acquisition starting from a 1 kHz GEO brick motion control box. The positions up to 3 kHz are captured by the Zebra box (blue and green acquisition points along the trajectory), beyond are interpolated post-acquisition (yellow points along the trajectory).

The up-triggering principle applied to the motion controller, can be further extended to the trigger box. Because the motors are moving along predictable trajectories, additional detector frames can be recorded, without position capture, in-between those requested by the trigger box. The missing positions are later interpolated from those captured. The detector implemented in the acquisition chain is an Eiger 500 k^21^, which can acquire multiple images per input trigger and has a maximum frame rate of 9 kHz for 30 s at 4-bit depth. By configuring the Eiger detector to acquire up to three images equally spaced in time per input trigger from the Zebra box, the final collection rate can be increased to 9 kHz. The schematic of the acquisition based on the two up-triggering steps is shown in Fig. 1.

### Parameter optimisation

During a continuous scanning acquisition, the detector records while the sample moves. The time during which the detector records is the exposure and the time between subsequent exposures is the acquisition. If the movement during the exposure is greater than the resolution of the system, the movement is perceived as a blurring of the image and a degradation of the data quality. In ptychography, spatial modes can be introduced to mitigate the effects of partial coherence caused by the movement^22^. As investigated elsewhere^14^, it is not trivial to define a relation between the optimum number of modes and experimental continuous scanning parameters. We estimated the number of modes required as the ratio between the distance travelled during the detector exposure time and the theoretical resolution given as the double of the reconstructed pixel size, such that $N_{{{\text{modes}}}} = \frac{\Delta x}{{2 d}}$, where *N*_modes_ is the number of modes, $\Delta x$ is the travelled distance and *d* the pixel size.

For a given ratio between the exposure and acquisition time, increasing the motor speed directly increases the number of modes required, along with the computational effort for the image reconstruction. A way to counterbalance this is to reduce the ratio between the exposure and the acquisition time. This approach is effective until entering the photon limited regime, where the resolution becomes limited by counting statistics. The optimal configuration for continuous scanning ptychography is a trade-off between resolution and speed, which varies depending on the sample and experimental conditions and is investigated in the following sections.

### 2D experiment

We apply the presented triggering scheme to image a Siemens star test pattern under different experimental conditions, motor speed and acquisition rate and duty cycle, to explore their effect on image resolution. The data were acquired at the I13-1 branchline of the Diamond Light source^23^. General experimental details can be found in the Methods section. The illumination size at the sample was 2.5 µm, the sample was scanned on a grid of 64 × 32 points with steps of 0.5 µm both horizontally and vertically, and the detector was placed 3.5 m downstream of the sample. All reconstructions were performed with 1000 iterations of the ePIE algorithm implemented in PtyREX^24^. The number of modes implemented in the reconstructions were based on the equation $N_{{{\text{modes}}}} = \frac{\Delta x}{{2{ }d}}$, as explained in the previous section. The parameters for the different scans are listed in Table 1.

**Table 1:** Parameters for the different ptychographic scans of the Siemens star test pattern.

Acquisition Frequency [Hz]	Exposure Time [ms]	Motor speed [µm/s]	Scanning speed [µm^2^/s]	Reconstructed pixel size [nm]	Modes	Resolution [nm]
100	5.0	50	25	23	6	56
100	3.0	50	25	23	3	55
200	3.0	100	50	23	6	58
833	1.0	417	210	46	5	169
1000	0.5	500	250	92	2	284
1000	0.1	500	250	92	1	404

**Figure 2:**
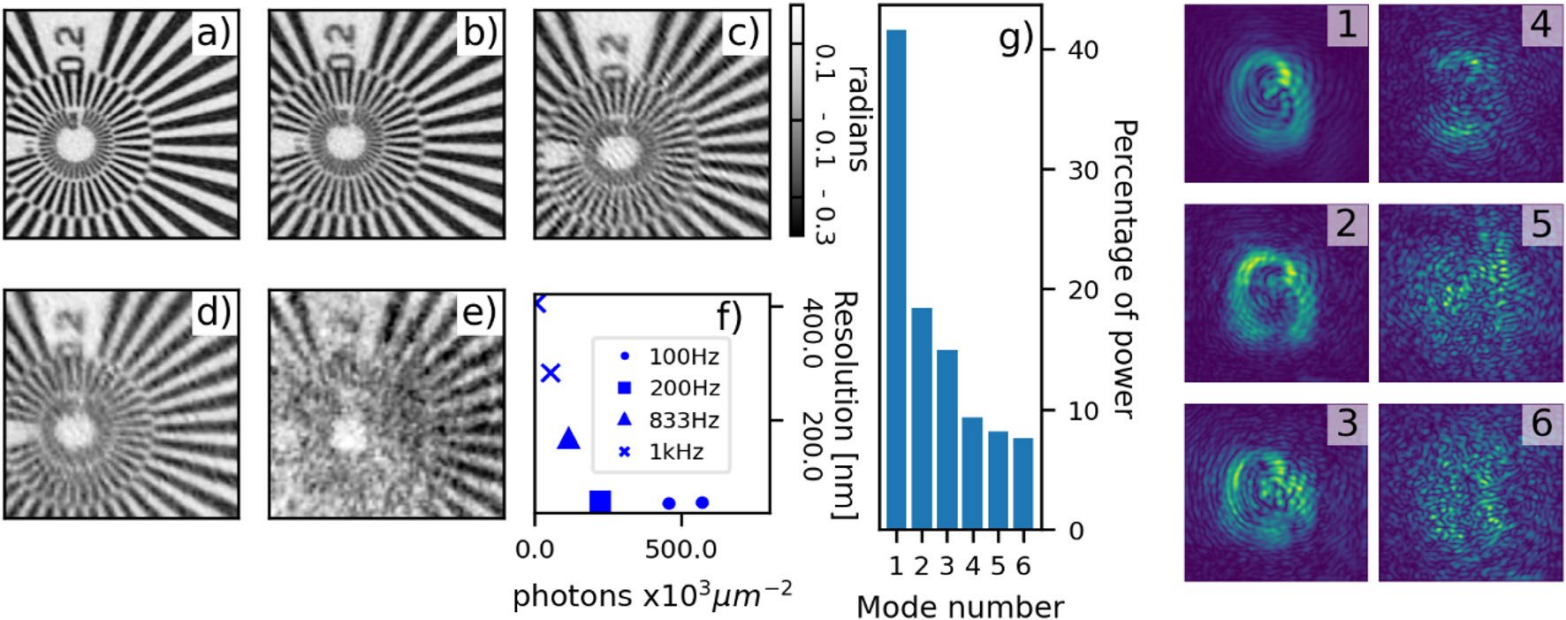
(**a**–**e**) Reconstructed phase images of the central part of the Siemens star test pattern (cropped to 6.9 × 6.9 μm) for different acquisition rates and exposure times. (**a**) 100 Hz, 5 ms, (**b**) 200 Hz, 3 ms, (**c**) 833 Hz 1 ms, (**d**) 1 k Hz 0.5 ms, (**e**) 1 kHz 0.1 ms. (**f**) Image resolution for the acquisition listed in Table 1 as a function of the photon density. (**g**) Percentage of power in the modes decomposition of (**a**). (1–6) Modulus of the probe modes reconstructions after orthogonalization.

Figure 2a–e shows the reconstructed phase object for the different parameters. As an example, we show the mode decomposition for the 100 Hz 5 ms acquisition: the occupancy and the structure of the modes can be seen in Fig. 2g. Figure 2f shows the resolution, measured with the Fourier ring correlation^25^, as a function of the photons per unit area. The resolution rapidly worsens for photon densities below 250 × 10^3^ μm^−2^ while it flattens above 300 × 10^3^ μm^-2^. The two 1 kHz measurements (squares in Fig. 2f) show a strong correlation between photon density and resolution, contrary to the two 100 Hz measurements (circles in Fig. 2f). This indicates that the 1 kHz measurements were in a photon limited regime.

To test the triggering method up to the maximum acquisition rate of the detector, while avoiding the photon starvation, the photon statistics is improved by decreasing the step size and widening the source defining slits. The latter lowers the coherence length and the ability to phase to high-resolution information, while increasing the flux for lower-resolution information.

**Figure 3:**
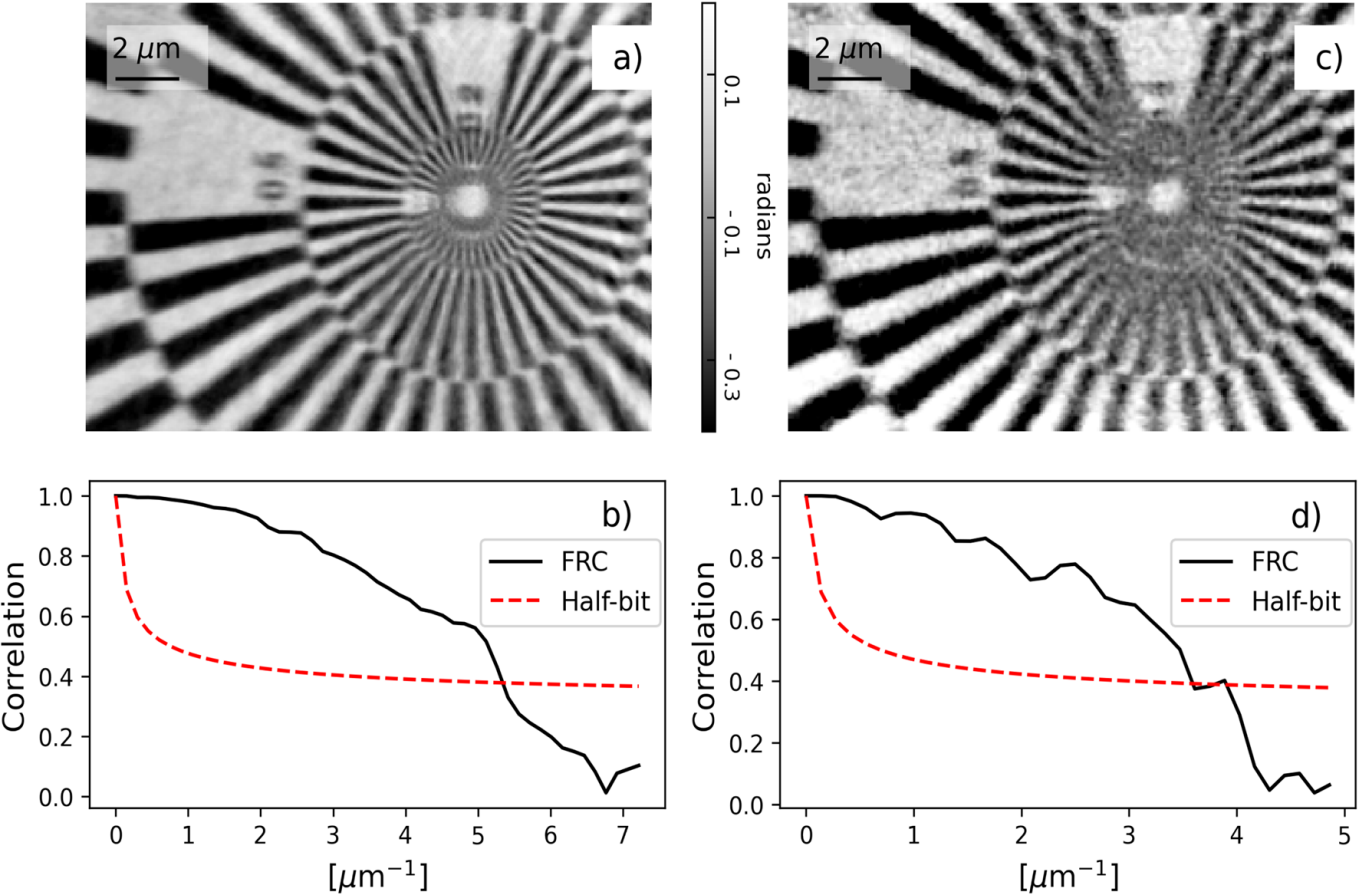
(**a**) Reconstructed phase object for the 2 kHz scan, pixel size 66.5 nm. (**b**) Fourier ring correction of (**a**) showing a resolution of 186 nm. (**c**) reconstructed phase object for the 9 kHz, pixel size 96 nm. (**d**) Fourier ring correction of (**c**) showing a resolution of 285 nm.

For the following set of acquisitions, the geometry of the experiment was changed with respect to that of the examples in Table 1. The illumination size at the sample was 6.2 μm, and the detector was 14.5 m downstream of the sample. The 3 × up-triggering scheme was used to approach 2 kHz (scan size 128 × 32, step size 0.2 × 1.0 μm, source size 50 μm, scanning frequency 1960 Hz, 500 ms exposure, 10 ms overhead) with a measured resolution of 186 nm (Fig. 3a,b). The 9 × up-triggering scheme was used to achieve a 9 kHz (scan size 576 × 16 step size 0.06 × 0.6 mμ, source size 200 μm, 78 ms exposure), with resolution of 285 nm (Fig. 3c,d). The difference in resolution for the 2 kHz and 9 kHz acquisitions is due to the different duty cycle, step size and source size. These combined together affect the photon statistics which was, for the 9 kHz scan, 2 times lower than for the 2 kHz.

### 3D experiment

The 2D triggering scheme was adapted to perform 3D ptychographic tomography by extending the PMAC motion program to also control the rotation stage. The maximum data writing rate of the Zebra (500 Hz) and its buffer size (~ 200 k points) limit the number of scan points that can be collected at its maximum rate (3 kHz). Full ptychographic tomography datasets require more points than the Zebra can store. Therefore, at rates larger than 500 Hz, the data is acquired and written in chunks, with N projections per chunk to avoid filling the memory buffer.

**Figure 4:**
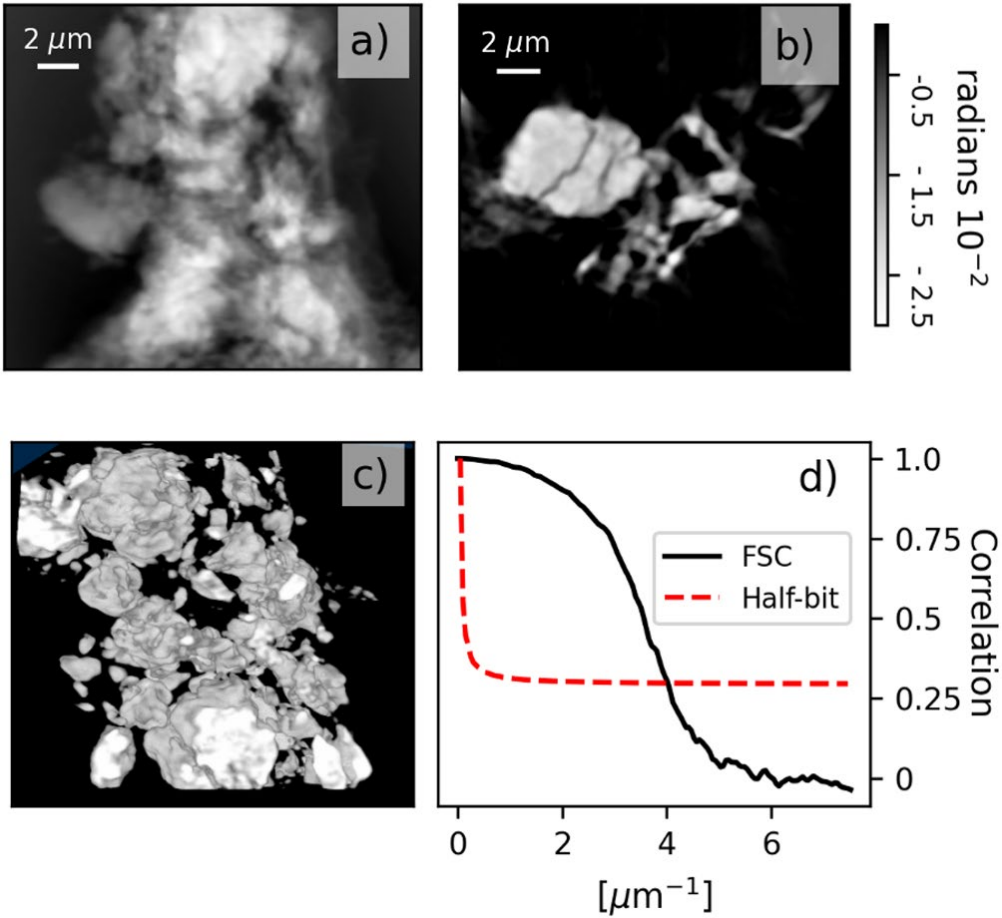
2 kHz ptychographic tomography scan reconstruction. (**a**) Reconstructed phase projection of NMC particle. (**b**) 2D slice of the 3D reconstructed volume. (**c**) 3D view of the reconstructed volume. (**d**) Fourier shell correlation for the 3D volume (resolution 250 nm).

The 3D acquisition scheme is used here to image a sample of NMC-811 battery cathode material. The 3D scan was performed acquiring 600 projections equally distributed over 180 degrees. Each projection was acquired with a 2D scan of 150 × 20 steps with increments of 0.24 μm and 1.0 µm, in the horizontal and vertical direction respectively. The frame rate was 2 kHz with an exposure time of 0.5 ms. The chunking size was set to 10 and the total 3D scanning time was 3 h due to the remaining overhead caused by the data writing. The reconstructed pixel size is 66.5 nm and 3D the resolution is 250 nm (see Fig. 4).

## Discussion

We have presented an acquisition scheme for ptychographic data collection in the kHz regime. The acquisition scheme allows to match the maximum acquisition rate of the detector with slower controller and trigger boxes with the up-triggering approach, moving the limiting factor from the motion system to the detector. We have successfully applied the scheme to perform 2D ptychographic scans at 9 kHz, with scanning speeds of up to 273 µm^2^/s at 285 nm resolution. We applied the method to 3D scanning by acquiring a ptychographic tomography at 2 kHz acquisition rate equivalent to 1.5 s per the projection and an isotropic resolution of 250 nm.

We performed an investigation on the dependency of the resolution on the photon density with a series of 2D scans. In the regime of hundreds of Hz the study shows that the same exposure at different acquisition rates and different exposures at the same acquisition rate produce similar resolutions. Both the total counts and the number of modes varied for each case, yet the resolution remained constant. This indicates that the experimental setup was limiting the resolution to approximately 50 nm. In the kHz regime, the resolution was limited by the photon statistics. It is not possible, with the acquired data, to fully decouple the effect of the increased motor speed and the photon starvation, which will require further investigation. The presented scheme was applied but it is not limited to snake-like trajectories. It is possible to implement different motion trajectories, such as spiral scans, by using the basic mathematical operators available for the PMAC scripts.

In summary, we have presented a multi kHz triggering scheme which shifts the limiting factor of a ptychographic scan from the motors to the detector and coherent flux. The 3D recording time is currently affected by overhead due to the data writing. Schemes of continuous readout at GB/s has already been successfully implemented^26^. Acquiring data using such a readout system would reduce the acquisition time for a 3D ptychography scan from hours to minutes. As an example, the acquisition time for the 2 kHz 3D scan presented in this work, would go from 3 h down to 15 min, and to 10 min at 3 kHz.

With the advent of the diffraction limited storage rings, the coherent photon flux increase will allow for shorter exposures before reaching the photon limited regime. The scalable approach outlined here applied at fourth generation light sources, will enable an increase in the data collection rate, to achieve higher resolutions at the current collection rates, or a combination of the two. The 9 kHz data presented here had an average of less than one count per pixel in the first order disk from the zone plate. Given the 4-bit pixel depth at this rate, there is still capacity for an order of magnitude increase in flux in this geometry at 3rd generation x-ray synchrotron beamlines like I13-1 of the Diamond Light Source. Taking full advantage of brighter light sources does require high speed detectors with larger bit depth than 4 bits. These detector developments are in progress and expected in the near future, as requested for x-ray imaging applications at diffraction-limited storage rings and XFELs.

## Methods

### Beamline setup

The beamline was operated in monochromatic mode using the Si111 double crystal monochromator at 9.7 keV. A high efficiency Fresnel zone plate lens, 400 µm diameter, 150 nm outermost width, was used to define the illumination at the sample. The sample was continuously scanned using a 3 axis PI-Mars nano-positioning stage, controlled via the GEO brick motion controller (Fig. 1).

The actual positions of the motors were read via the encoders connected to the Zebra trigger box with a frequency up to 3 kHz. For acquisition greater than 3 kHz, the detector was acquiring up to 2 extra images per Zebra trigger. The corresponding positions were interpolated from those measured with the encoders. In the reconstruction, the annealing method^27^ is applied for the position correction, to deal with possible difference between the interpolated and actual positions of the motors during the detector exposure.

### Detector

The detector used for recording the diffraction pattern in the far field is an Eiger X 500 k photon counting detector with energy range 5 keV to 36 keV. The sensor material is 450 µm thick Si, with 75 µm pixel size, and 1030 pixel × 514 pixel active area. The readout bit depth is 12 bit up to 3 kHz, 8 bit between 3 and 8 kHz and 4 bit from 8 to 9 kHz. The detector is capable on 3 kHz continuous acquisition and 9 kHz in burst mode up to 30 s acquisition. The detector can be operated in external trigger mode, and can acquire multiple images per received external trigger, equally spaced in time.

### Sample

The sample used for the 2D scans is a Siemens star test pattern, 250 nm thick gold deposited on a silicon nitride membrane, with inner separation of the spoke of 50 nm.

The 3D sample is an NMC-811 powder from battery cathode material.

## Data Availability

The authors confirm that all of the data used in this study are available without restriction. Data can be obtained by contacting darren.batey@diamond.ac.uk.

## References

[1] Rodenburg, J. M. *et al.* Hard-x-ray lensless imaging of extended objects. *Phys. Rev. Lett.* **98** (2007). 10.1103/PhysRevLett.98.034801 17358687

[2] Maiden AM, Rodenburg JM (2009). An improved ptychographical phase retrieval algorithm for diffractive imaging. Ultramicroscopy.

[3] Guizar-Sicairos M, Fienup JR (2008). Phase retrieval with transverse translation diversity: a nonlinear optimization approach. Opt. Express.

[4] Enders, B. & Thibault, P. in *Proceeding of the Royal Society A.* (2016).

[5] Marchesini S (2016). SHARP: a distributed GPU-based ptychographic solver. J. Appl. Crystallogr..

[6] Donnelly C (2017). Three-dimensional magnetization structures revealed with X-ray vector nanotomography. Nature.

[7] Diaz A (2015). Three-dimensional mass density mapping of cellular ultrastructure by ptychographic X-ray nanotomography. J. Struct. Biol..

[8] Holler M (2019). Three-dimensional imaging of integrated circuits with macro- to nanoscale zoom. Nature Electronics.

[9] Odstrcil M, Lebugle M, Lachat T, Raabe J, Holler M (2019). Fast positioning for X-ray scanning microscopy by a combined motion of sample and beam-defining optics. J. Synchrotron Radiat..

[10] Pelz PM (2014). On-the-fly scans for X-ray ptychography. Appl. Phys. Lett..

[11] Deng J (2019). The Velociprobe: An ultrafast hard X-ray nanoprobe for high-resolution ptychographic imaging. Rev. Sci. Instrum..

[12] Deng J (2015). Continuous motion scan ptychography: characterization for increased speed in coherent x-ray imaging. Opt. Express.

[13] Clark JN, Huang X, Harder RJ, Robinson IK (2014). Continuous scanning mode for ptychography. Opt. Lett..

[14] Huang X (2015). Fly-scan ptychography. Sci. Rep..

[15] Odstrčil M, Holler M, Guizar-Sicairos M (2018). Arbitrary-path fly-scan ptychography. Opt. Express.

[16] Jones MWM (2022). High-speed free-run ptychography at the Australian Synchrotron. J. Synchrotron Radiat..

[17] Jiang Y (2021). Achieving high spatial resolution in a large field-of-view using lensless x-ray imaging. Appl. Phys. Lett..

[18] Cobb, T. M., Chernousko, Y. S. & Uzun, I. S. in *ICALEPCS'13.* 736–739.

[19] Sherrell DA (2015). A modular and compact portable mini-endstation for high-precision, high-speed fixed target serial crystallography at FEL and synchrotron sources. J. Synchrotron Radiat..

[20] Owen RL (2017). Low-dose fixed-target serial synchrotron crystallography. Acta. Crystallogr. D Struct. Biol..

[21] Tinti G (2015). Performance of the EIGER single photon counting detector. J. Instrum..

[22] Li P, Edo T, Batey D, Rodenburg J, Maiden A (2016). Breaking ambiguities in mixed state ptychography. Opt. Express.

[23] Rau C (2017). Imaging with Coherent Synchrotron Radiation: X-ray Imaging and Coherence Beamline (I13) at Diamond Light Source. Synchrotron Radiat. News.

[24] Batey, D. J. *Ptychographic Imaging of Mixed States*, University of Sheffield, (2014).

[25] Koho S (2019). Fourier ring correlation simplifies image restoration in fluorescence microscopy. Nat. Commun..

[26] Marone F, Studer A, Billich H, Sala L, Stampanoni M (2017). Towards on-the-fly data post-processing for real-time tomographic imaging at TOMCAT. Adv. Struct. Chem. Imaging.

[27] Maiden AM, Humphry MJ, Sarahan MC, Kraus B, Rodenburg JM (2012). An annealing algorithm to correct positioning errors in ptychography. Ultramicroscopy.

